# Supplementary material for: ATF3 acts as a rheostat to control JNK signalling during intestinal regeneration
Source: Nat Commun. 2017 Mar 8;8:14289. doi: 10.1038/ncomms14289 (PMC5344978; doi:10.1038/ncomms14289)
Supplement: Supplementary Information — Supplementary Figures, Supplementary Tables and Supplementary References [file ncomms14289-s1.pdf]

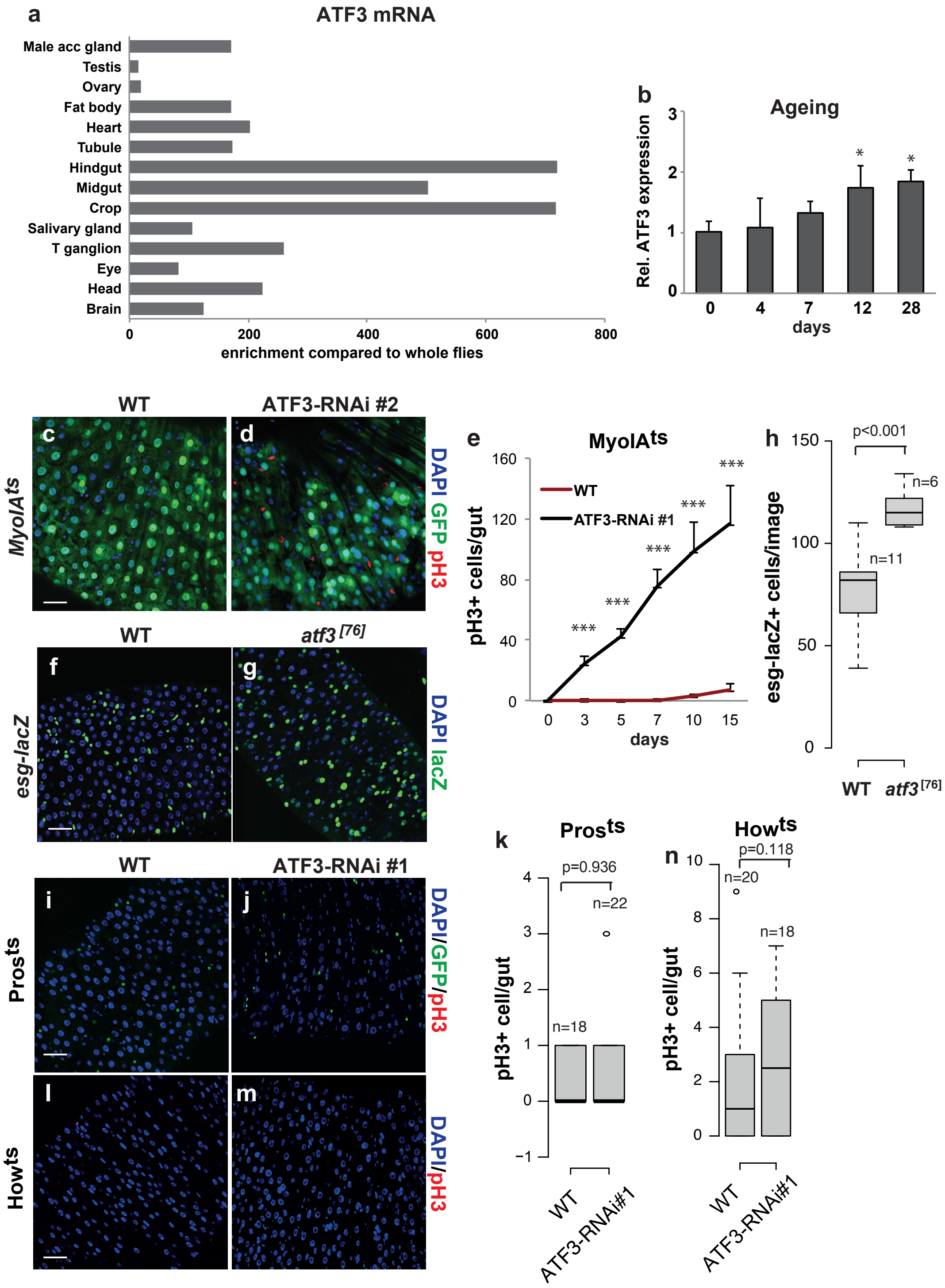

a
ATF3 mRNA
Male acc gland
Testis
Ovary
Fat body
Heart
Tubule
Hindgut
Midgut
Crop
Salivary gland
T ganglion
Eye
Head
Brain
0 200 400 600 800
enrichment compared to whole flies
b
Ageing
Rel. ATF3 expression
0 1 2 3
0 4 7 12 28
days
WT
ATF3-RNAi #2
c
d
MyoIAts
DAPI GFP pH3
e
MyoIAts
WT
ATF3-RNAi #1
pH3+ cells/gut
0 40 80 120 160
0 3 5 7 10 15
days
h
p<0.001
n=6
n=11
esg-lacZ+ cells/image
0 50 100 150
WT atf3[76]
WT
atf3[76]
f
g
esg-lacZ
DAPI lacZ
k
Prosts
p=0.936
n=22
n=18
pH3+ cell/gut
n
Howts
p=0.118
n=20
n=18
pH3+ cell/gut
WT
ATF3-RNAi #1
i
j
Prosts
DAPI/GFP/pH3
l
m
Howts
DAPI/pH3
WT
ATF3-RNAi#1

Supplementary Figure 1. ATF3 is enriched in the intestine and restricts stem cell proliferation

(a) Enrichment of ATF3 expression in the digestive tract of *Drosophila*. Expression is shown as a ratio of mRNA enrichment for each gene in each tissue to the average mRNA enrichment for all the tissues (data from FlyAtlas, Chintapalli et al., 2007). (b) Expression of ATF3 in the ageing female intestine measured by RT-qPCR. Shown are normalized values. P values (* p<0.05; ** p<0.01; *** p<0.001) from Student's t-test are shown in b. Mean fold change ±s.e. based on three replicated experiments. (c) Control adult female midgut-*MyoIA^{ts}>w1118* (WT) and (d) ATF3 RNAi lines-*MyoIA^{ts}>ATF3-RNAi #2* (TRIP library) expressed the female midgut after 7 days at 29°C. Stained for pH3 (red) and DNA (blue) (e) Quantification of pH3 positive cells per adult midgut in ageing female flies of the indicated genotypes. Mean ±s.e. P values (* p<0.05; ** p<0.01; *** p<0.001) were obtained by Student's t-test. 12 midguts were used in one experiment, which was repeated three times, is shown in e. Wild-type male midgut (f) displayed fewer *esg-lacZ* expressing cells compared to *atf3$^{[76]}$* mutant male(g). (h) Quantification of *esg-lacZ* positive cells per adult midgut of the indicated genotypes after 3 days at 25°C. (i-j) The representative image of posterior midgut of female *Pros^{ts}>WT* (i) or *Pros^{ts}>ATF3 RNAi#1* (j) after 7 days at 29°C, stained with pH3 antibody in red. (k) Quantification of pH3 positive cells per adult female midgut of the indicated genotypes. (l-m) The representative image of posterior midgut of female *How^{ts}>WT* (l) or *How^{ts}>ATF3 RNAi#1* (m) after 7 days at 29°C, stained with pH3 antibody in red. (n) Quantification of pH3 positive cells per adult midgut of the indicated genotypes. P values from Student's t-test are shown in h, k and n. Mean ±s.e. Numbers of guts scored for each genotype are indicated from three replicated experiments are shown in h, k and n. Scale bars: 30 μm (c-d, f-g, i-j, l-m).

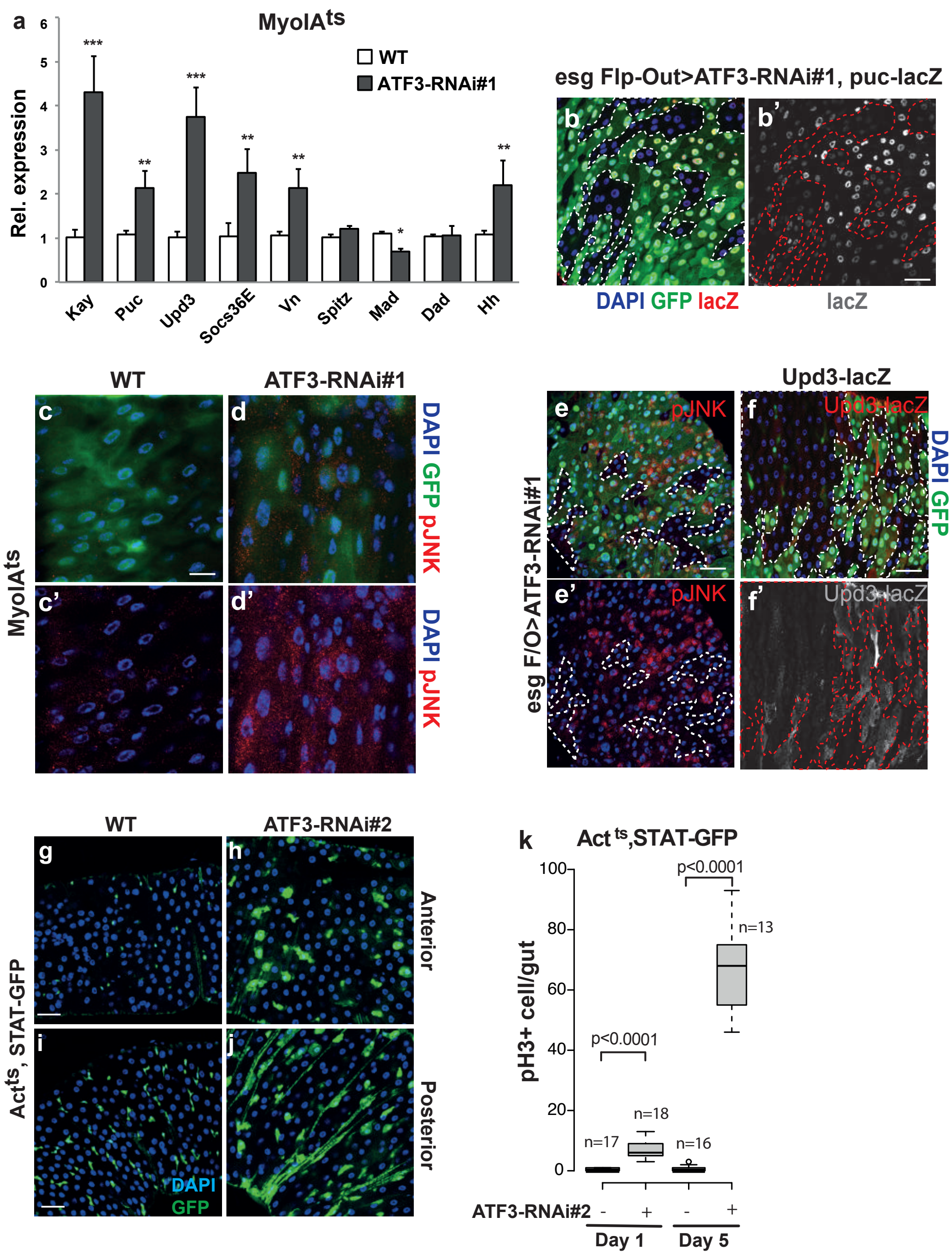
a
MyoIAts
WT
ATF3-RNAi#1
Rel. expression
Kay
Puc
Upd3
Socs36E
Vn
Spitz
Mad
Dad
Hh
esg Flp-Out>ATF3-RNAi#1, puc-lacZ
b
b'
DAPI GFP lacZ
lacZ
WT
ATF3-RNAi#1
c
d
c'
d'
MyoIAts
DAPI GFP pJNK
DAPI pJNK
Upd3-lacZ
e
f
e'
f'
pJNK
Upd3-lacZ
esg F/O>ATF3-RNAi#1
DAPI GFP
WT
ATF3-RNAi#2
g
h
i
j
Anterior
Posterior
Actts, STAT-GFP
DAPI
GFP
k
Act ts,STAT-GFP
pH3+ cell/gut
p<0.0001
p<0.0001
n=17
n=18
n=16
n=13
ATF3-RNAi#2
Day 1
Day 5

Supplementary Figure 2. Activation of JNK and STAT signaling upon loss-of ATF3

(a) RT-qPCR analysis of the female intestine shows that the expression levels of JNK (*Kay*, *Puc*), JAK–STAT (*Upd3*, *Socs36E*) and EGFR (*Vein*) ligands/target genes were increased upon enterocyte-specific knock down of ATF3 after 7 days at 29°C. Mean fold change ±s.e. based on three replicated experiments. P values from Student's t-test (* $p<0.05$; ** $p<0.01$; *** $p<0.001$) are shown. (b) *puc-lacZ* expression (red in b, grey in b') in esg^{ts} *F/O* induced *ATF3 RNAi* cell clones after 2 days at 29°C. (c-d) Activation of JNK signaling in ATF3 depleted female midgut ($MyoIA^{ts}$*>ATF3-RNAi*, panel d) by phospho-JNK staining (red) after 3 days at 29°C, compared to WT midgut ($MyoIA^{ts}$*>WT*, panel c). (e) Activation of JNK signaling (phosphor-JNK staining in red, panel e and e') in esg^{ts} *F/O* induced *ATF3 RNAi* cell clones after 2 days at 29°C. (f) *Upd3-lacZ* expression (beta-galactosidase staining in red, panel f and f') in esg^{ts} *F/O* induced *ATF3 RNAi* cell clones. (g-i) After shift to 29°C for one day, Act^{ts}*>ATF3-RNAi* (h, j) midguts showed elevated JAK/STAT activity (10X*STAT-GFP*), compared to WT control (Act^{ts}*>WT*, panel g and i). (k) pH3 Quantification per midgut of flies with indicated genotypes ($MyoIA^{ts}$*>WT*, $MyoIA^{ts}$*>ATF3-RNAi#2* at 29°C for 1 day and 5 days, respectively). Scale bars: 30 µm (b, e-j) and 10 µm (c-d).

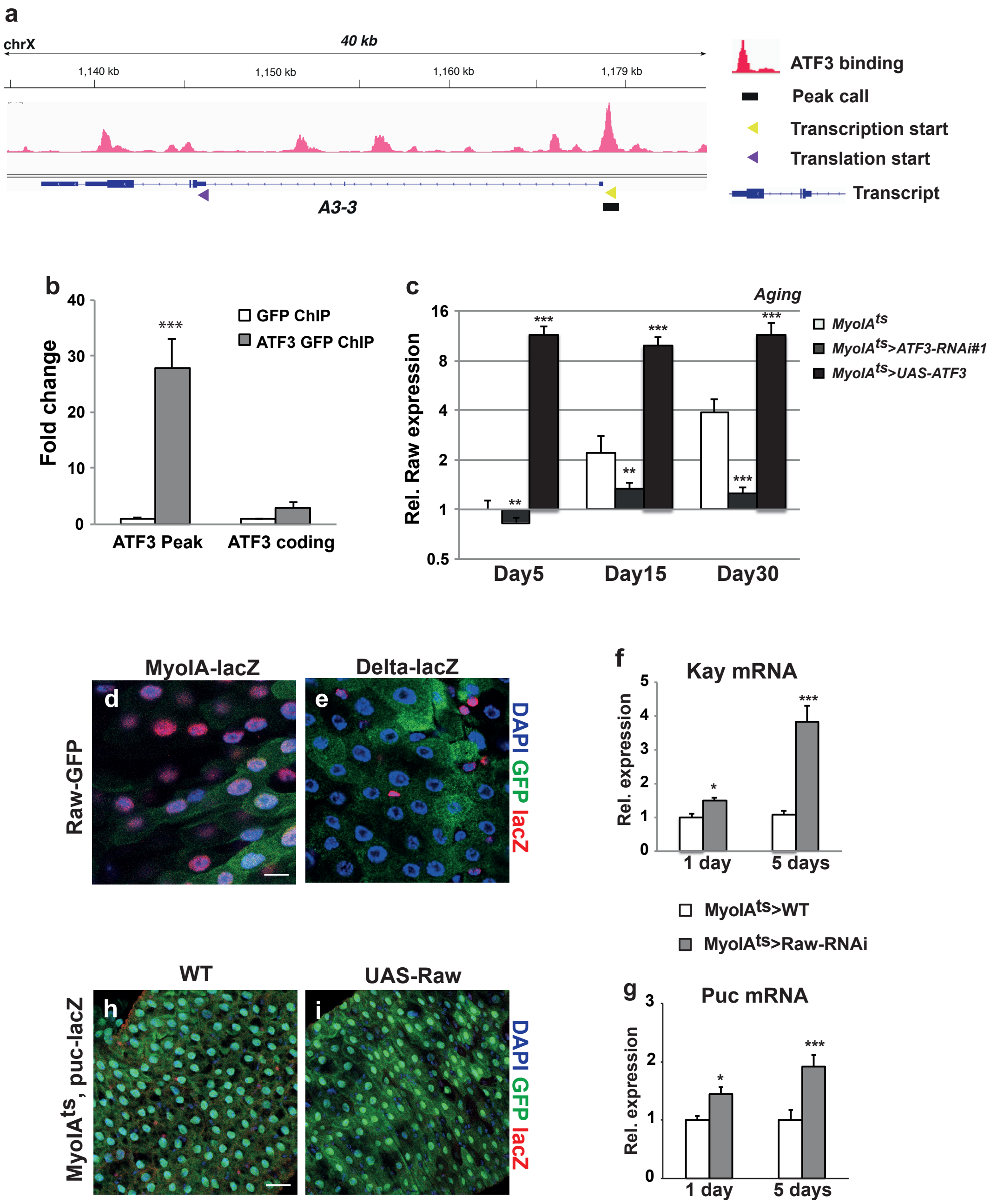

a
chrX
40 kb
1,140 kb
1,150 kb
1,160 kb
1,179 kb
A3-3
ATF3 binding
Peak call
Transcription start
Translation start
Transcript
b
Fold change
GFP ChIP
ATF3 GFP ChIP
ATF3 Peak
ATF3 coding
c
Aging
Rel. Raw expression
MyoIA^ts
MyoIA^ts>ATF3-RNAi#1
MyoIA^ts>UAS-ATF3
Day5
Day15
Day30
MyoIA-lacZ
Delta-lacZ
Raw-GFP
d
e
DAPI GFP lacZ
f
Kay mRNA
Rel. expression
1 day
5 days
MyoIA^ts>WT
MyoIA^ts>Raw-RNAi
WT
UAS-Raw
MyoIA^ts, puc-lacZ
h
i
DAPI GFP lacZ
g
Puc mRNA
Rel. expression
1 day
5 days

Supplementary Figure 3. ATF3 transcriptionally targets itself and Raw expression in enterocytes.

(a) ChIP-seq track for ATF3-GFP protein at the *ATF3* (A3-3) locus. Black block represents bound region (peak enriched region). ATF3 binds at its own promoter. P values (* p<0.05; ** p<0.01; *** p<0.001, Student's *t*-test) are shown in b and c. Mean fold change ±s.e. based on three replicated experiments. (b) ChIP-qPCR analysis at *ATF3* locus in midgut of ATF3::GFP female flies compared to WT midgut. A region in the *ATF3*-coding sequence was selected as negative control. (c) Expression of *Raw* in control (*MyoIA^{ts}>w1118*), ATF3 knock down (*MyoIA^{ts}>ATF3-RNAi*), and ATF3 overexpression (*MyoIA^{ts}>UAS-ATF3*) female flies at 29°C for 5, 15 and 30 day after RNAi induction. Mean fold change ±s.e. based on three replicated experiments. (d) Beta-galactosidase staining (red) in the intestine of *Raw-GFP>MyoIA-lacZ* flies revealed that Raw-GFP is expressed in MyoIA-lacZ positive cells. (e) Beta-galactosidase staining (red) in the intestine of *Raw-GFP>Delta-lacZ* flies revealed that Raw-GFP is not expressed in Delta-lacZ positive cells. (f, g) RT-qPCR assay of JNK components *Kay* (f), *Puc* (g) expression on gut extracts of WT and *MyoIA^{ts}>Raw-RNAi* female midguts at 1 and 5 days after RNAi induction. The significant differences in gene expression between each RNAi group (*MyoIA^{ts} >Raw-RNAi*) and the control group (*MyoIA^{ts} >w1118*) are indicated with asterisks (* p<0.05; ** p<0.01; *** p<0.001, Student's t-test). Mean ±s.e. based on three replicated experiments are shown in f and g. (h-i) The representative image of posterior midgut epithelium of WT (*MyoIA^{ts}, Puc-lacZ>w1118*) and Raw expression female flies (*MyoIA^{ts}, Puc-lacZ >UAS-Raw*) by Beta-galactosidase staining in red, and stained for DNA in blue with *MyoIA-GFP* (green), the transgene was expressed at 29°C for 5 days. Scale bars: 30μm (h-i), 10μm (d-e).

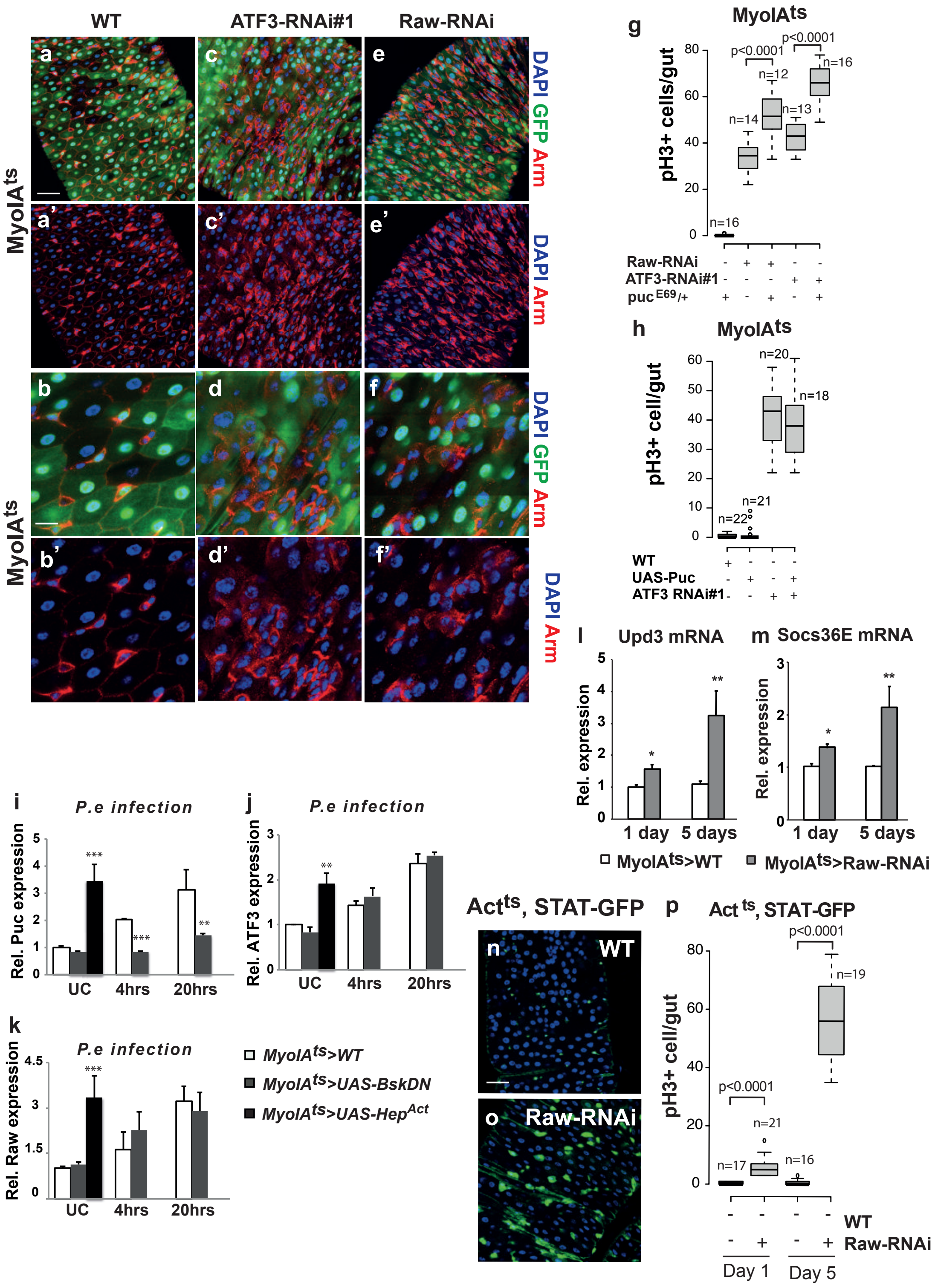
WT
ATF3-RNAi#1
Raw-RNAi
MyoIAts
a
c
e
a'
c'
e'
b
d
f
b'
d'
f'
DAPI GFP Arm
DAPI Arm
g
MyoIAts
pH3+ cells/gut
p<0.0001
p<0.0001
n=16
n=14
n=12
n=13
n=16
Raw-RNAi
ATF3-RNAi#1
puc E69/+
h
MyoIAts
pH3+ cell/gut
n=20
n=18
n=22
n=21
WT
UAS-Puc
ATF3 RNAi#1
l
Upd3 mRNA
m
Socs36E mRNA
Rel. expression
1 day
5 days
MyoIAts>WT
MyoIAts>Raw-RNAi
i
P.e infection
Rel. Puc expression
UC
4hrs
20hrs
j
P.e infection
Rel. ATF3 expression
k
P.e infection
Rel. Raw expression
MyoIAts>WT
MyoIAts>UAS-BskDN
MyoIAts>UAS-HepAct
Actts, STAT-GFP
n
WT
o
Raw-RNAi
p
Act ts, STAT-GFP
p<0.0001
p<0.0001
n=19
n=21
n=17
n=16
pH3+ cell/gut
WT
Raw-RNAi
Day 1
Day 5

Supplementary Figure 4. Raw is required for intestinal homeostasis.

(a-f) Representative image of *MyoIA*^{ts}*>w1118* (a-b), *MyoIA*^{ts}*>ATF3-RNAi#1* (c-d) and *MyoIA*^{ts}*>Raw-RNAi* (e-f) female midguts stained with apical-basal marker Armadillo. MyoIA-GFP positive cells are in green, nuclei are stained with DAPI (blue) and Armadillo positive cells are in red. (g) Quantification of pH3-positive cells per adult female midgut of the indicated genotypes (*MyoIA*^{ts}*>WT and Puc*^{E69}/+, *MyoIA*^{ts}*>Raw-RNAi*, *MyoIA*^{ts}*>Raw-RNAi and Puc*^{E69}/+, *MyoIA*^{ts}*>ATF3-RNAi#1, MyoIA*^{ts}*>ATF3-RNAi#1 and Puc*^{E69}/+, respectively) after shift to 29°C for 5 days. (h) Quantification of pH3-positive cells per adult midgut of the indicated genotypes (*MyoIA*^{ts}*>WT*, *MyoIA*^{ts}*>UAS-Puc, MyoIA*^{ts}*>ATF3-RNAi#1*, *MyoIA*^{ts}*>ATF3-RNAi#1 and UAS-Puc*, respectively) after shift to 29°C for 5 days. P values from Student's t-test are shown in g and h. Mean ±s.e. Numbers of guts scored for each genotype are indicated from three replicated experiments. (i-k) RT-qPCR assay of *Puc* (i), *ATF3* (j), and *Raw* (k) expression on gut extracts of *MyoIA*^{ts}*>WT*, *MyoIA*^{ts}*>UAS-Hep*^{Act} and *MyoIA*^{ts}*>UAS-Bsk*^{DN} female midguts in unchallenged (UC) and *P.e* infection (4, 16 hours) condition, the flies were shift to 29°C for 5 days before infection. (l-m) RT-qPCR assay of STAT components *Upd3* (l), *Socs36E* (m) expression on gut extracts of WT and *MyoIA*^{ts}*>Raw-RNAi* midguts at 1 and 5 days after RNAi induction. P values (* $p<0.05$; ** $p<0.01$; *** $p<0.001$, Student's *t*-test) are shown in i-m. Mean fold change ±s.e. based on three replicated experiments. (n-o) After shift to 29°C for 1 day, *Act*^{ts}*>WT* (n) and *Act*^{ts}*>Raw-RNAi* (o) anterior midguts carrying a GFP reporter for JAT/STAT activity (*STAT-GFP*). (p) Quantification of pH3-positive cells per adult female midgut of the indicated genotypes (*MyoIA*^{ts}*>WT* and *MyoIA*^{ts}*>Raw-RNAi* female midguts) after shift to 29°C for 1 and 5 days. Mean ±s.e. Numbers of guts scored for each genotype are indicated from three replicated experiments. The scale bar represents 30 µm (a, c, e, n-o), 10µm (b, d, f).

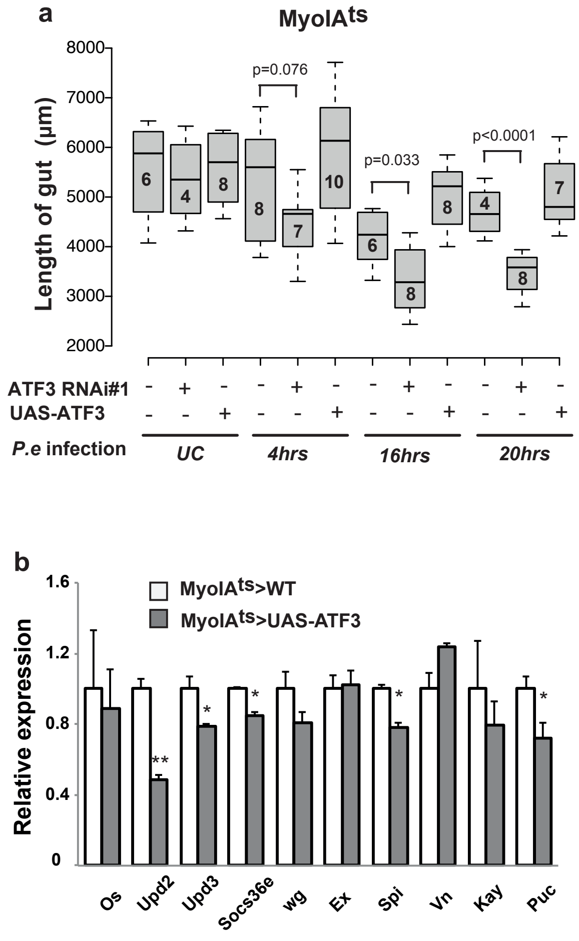

Supplementary Figure 5. ATF3 depletion leads to shrunken gut phenotype.

(a) Quantification of the length of female intestine in response to *P.e* infection for 4, 16 and 20 hours (hrs) using NeuronJ, an ImageJ plugin. The number shown in the box indicates number of midguts used in the experiment. The significant differences in the length of intestine between RNAi group (*MyoIA*^{ts} *>ATF3-RNAi#1*) and the control group (*MyoIA*^{ts} *>WT*) are indicated by p value (Student's *t*-test). (b) RT-qPCR analysis of the intestine shows that the expression levels of JNK (*Puc*), JAK–STAT (*Upd2*, *Upd3*, *Socs36E*) and EGFR (*Spi*) ligands/target genes were decreased upon enterocytes-specific overexpression of ATF3 in female flies for 7 days at 29°C. P values (* $p<0.05$; ** $p<0.01$; *** $p<0.001$, Student's *t*-test) are shown in b and c. Mean fold change ±s.e. based on three replicated experiments.

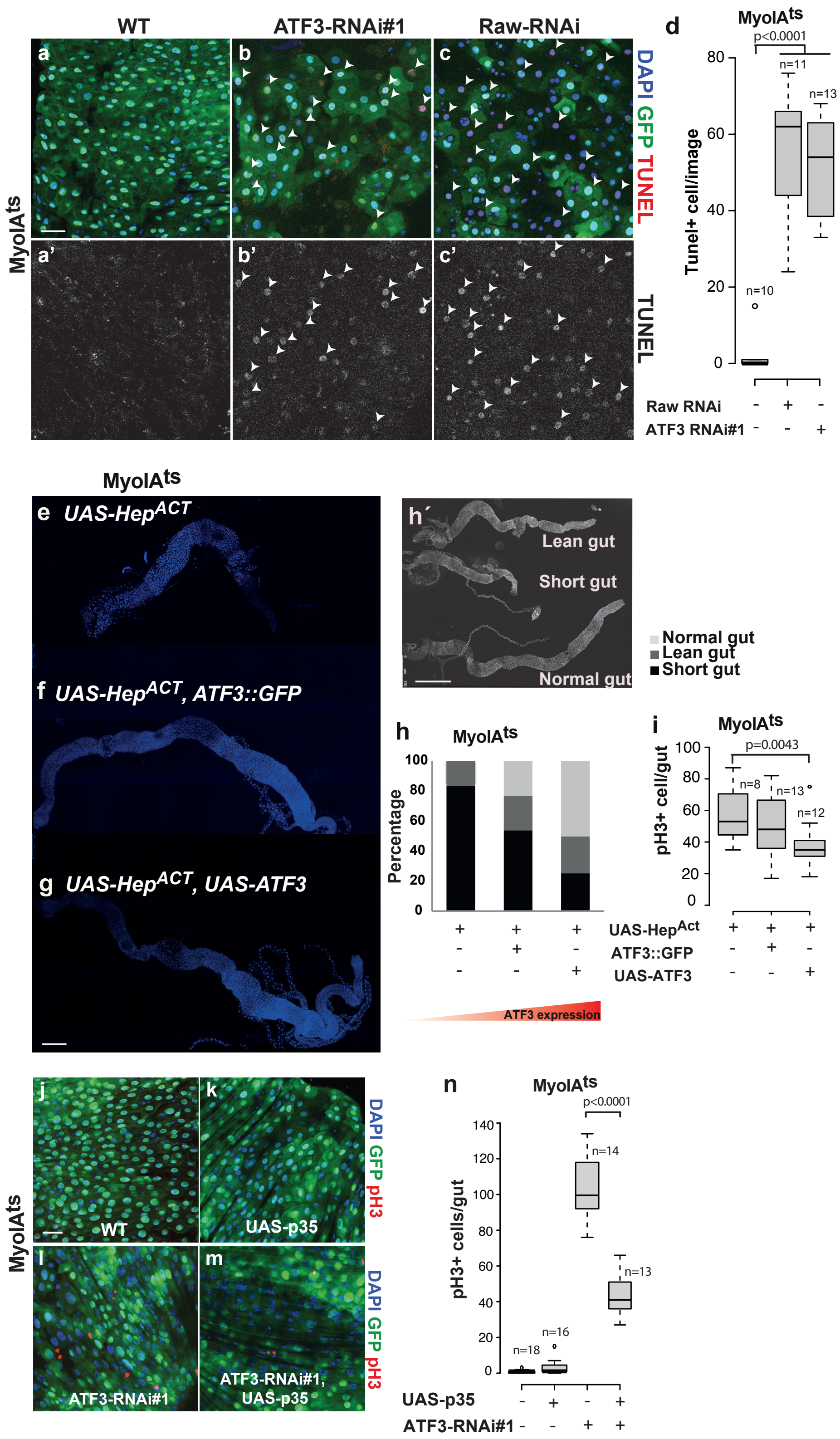

WT
ATF3-RNAi#1
Raw-RNAi
a
b
c
a'
b'
c'
DAPI GFP TUNEL
TUNEL
MyoIAts
d
MyoIAts
p<0.0001
Tunel+ cell/image
n=10
n=11
n=13
Raw RNAi
ATF3 RNAi#1
e UAS-HepACT
f UAS-HepACT, ATF3::GFP
g UAS-HepACT, UAS-ATF3
h´
Lean gut
Short gut
Normal gut
Normal gut
Lean gut
Short gut
h
MyoIAts
Percentage
ATF3 expression
i
MyoIAts
p=0.0043
pH3+ cell/gut
n=8
n=13
n=12
UAS-HepAct
ATF3::GFP
UAS-ATF3
j
k
l
m
WT
UAS-p35
ATF3-RNAi#1
ATF3-RNAi#1, UAS-p35
DAPI GFP pH3
n
MyoIAts
p<0.0001
pH3+ cells/gut
n=18
n=16
n=14
n=13
UAS-p35
ATF3-RNAi#1

Supplementary Figure 6. ATF3-Raw prevents JNK induced apoptosis.

(a-c) *Drosophila* midgut TUNEL assay. Condensed TUNEL positive nuclei (yellow arrowheads) are observed in *ATF3 RNAi* (b) and *Raw RNAi* (c) female midguts, indicated apoptosis is induced in ATF3 and Raw depleted midguts. No apoptotic cells are presented in Wild-type midguts (a). (d) Quantification of TUNEL positive cells per image with indicated genotypes (*MyoIA^{ts}>WT*, *MyoIA^{ts}>Raw-RNAi,* *MyoIA^{ts}>ATF3-RNAi,* respectively) shifted to 29°C for 4 days. P values from Student's t-test are shown. Mean ±s.e. Numbers of images are taken for the analysis for each genotype, which are indicated from three replicated experiments. (e) Expression of *Hep^{Act}* in EC causes dramatic change in gut morphology by inducing apoptosis. Representative images of intestines from *MyoIA^{ts}>UAS-Hep^{Act}*. (f-g) Additional copy of ATF3 (ATF3::GFP, panel f) or expression of ATF3 (g) in the female intestine of *MyoIA^{ts}>UAS-Hep^{Act}* displays normal gut morphology. Representative image of intestines from *MyoIA^{ts}>UAS-Hep^{Act}, ATF3::GFP* (f) and *MyoIA^{ts}>UAS-Hep^{Act}*, *UAS-ATF3* (g). (h) Morphology changes in the intestine of indicated genotype (*MyoIA^{ts}>UAS-Hep^{Act}*, *MyoIA^{ts}>UAS-Hep^{Act} and ATF3-GFP/+, MyoIA^{ts}>UAS-Hep^{Act} and UAS-ATF3,* respectively). After 4 days shift to 29°C. (i) pH3 Quantification per midgut of female flies with indicated genotypes (*MyoIA^{ts}>UAS-Hep^{Act}*, *MyoIA^{ts}>UAS-Hep^{Act} and ATF3-GFP/+, MyoIA^{ts}>UAS-Hep^{Act} and UAS-ATF3,* respectively) shifted to 29°C for 4 days. (j-m) The representative image of pH3 staining in the posterior midguts of female flies (*MyoIA^{ts}>ATF3-RNAi and UAS-p35*, *MyoIA^{ts}>UAS-p35, MyoIA^{ts}>ATF3-RNAi, MyoIA^{ts}>WT*). (n) pH3 Quantification per midgut of flies with indicated genotypes shifted to 29°C for 7 days. P values from Student's t-test are shown in i and n. Mean ±s.e. Numbers of guts scored for each genotype are indicated from three replicated experiments. Scale bars: 250 µm (e-g), 750 µm (f′), 30 µm (a-c, j-m)

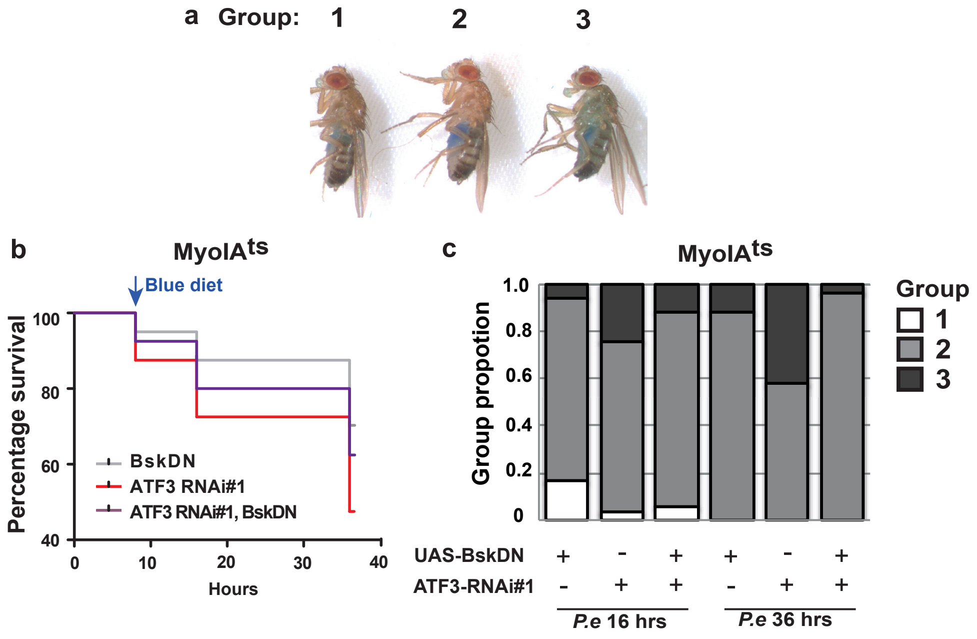

Supplementary Figure 7. JNK suppression prevents ATF3 loss induced barrier dysfunction and susceptibility to infection

(a) Representative images of 'Smurf' flies, representing different amount of blue dye in the hemolymph and divided into 1, 2 and 3 groups. (b) A parallel survival analysis of flies orally infected with the pathogenic bacteria *P.e*. Female llies were fed with *P.e* diet (5% sucrose) for 8 hrs and transferred to blue *P.e* diet and survival rate is monitored at 8hrs, 16hrs and 36hrs respectively. (c) The proportion of Smurf flies in each group following 8 hours or 24 hours transfer to blue food. n=40 flies per genotype per experiment. 3 independent experiments were performed.

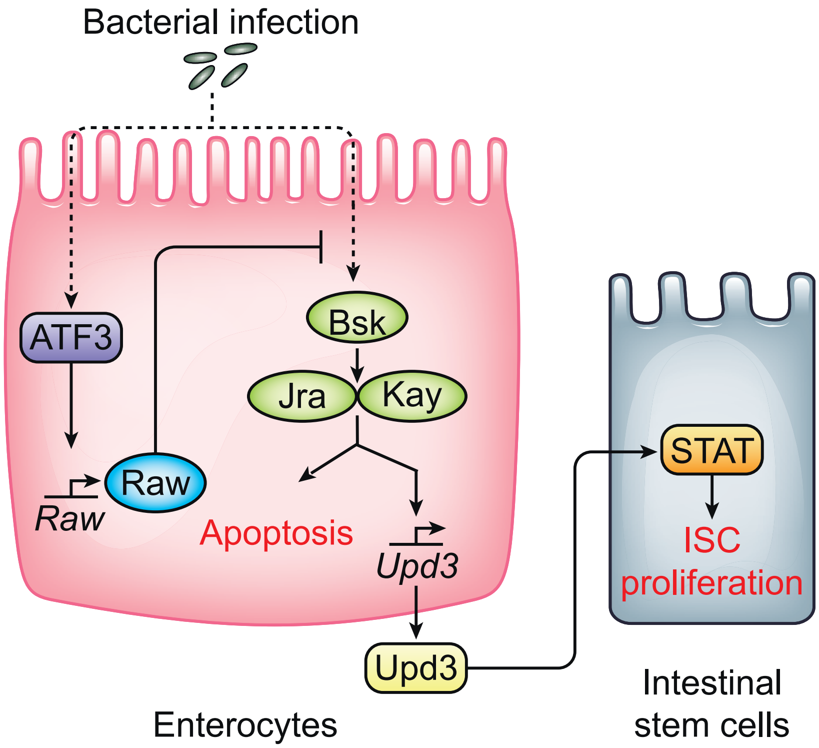

Supplementary Figure 8: Model for ATF3 in intestinal homeostasis

Supplementary Table 1: Mutant alleles used in the study

Mutant	Gene name	Type of mutation	Ref./ Source
atf3^{76}	Activating transcription factor 3	ATF3 bZIP domain knock-out by imprecise excision	1
Puc^{E69}	Puckered	Gene disruption by lacZ enhancer trap insertion	2
PGRP-LC^{7457}	Peptidoglycan recognition protein LC	EMS induced mutation	Akira Goto

Supplementary Table 2: Sequence information for qPCRs and ChIP-qPCRs

Name	Primer direction	Sequences	Probe No.
**ATF3**	Left	cagtgtctcacctgctggaa	125
**ATF3**	Right	gacaatcagatgggcagaact	125
**Dad**	Left	gagtcggttaccactgatgga	14
**Dad**	Right	ttttggcgtgaaagaactcc	14
**Kay**	Left	cagcatcagcgacaggatta	113
**Kay**	Right	tctggccggtctcaaagtt	113
**Mad**	Left	gaagttgaagaagcgcaagg	17
**Mad**	Right	ctgaccgggacaggagag	17
**Puc**	Left	cgtcatcatcaacggcaat	70
**Puc**	Right	aggcggggtgtgtttctat	70
**Raw**	Left	tcctgggtgacgagaagc	147
**Raw**	Right	tggccaagtcgatcaggta	147
**Rp49**	Left	ttccttgacgtgccaaaact	159
**Rp49**	Right	aatgatctataacaaaatcccctga	159
**Socs36E**	Left	aaaaagccagcaaaccaaaa	43
**Socs36E**	Right	aggtgatgacccattggaag	43
**spitz**	Left	gcgggtgtttttgttgtcat	31
**spitz**	Right	ttggaatcgggtttctctaca	31
**Upd3**	Left	cccagccaacgatttttatg	165
**Upd3**	Right	tgttaccgctccggctac	165
**Vn**	Left	tcacacatttagtggtggaagc	100
**Vn**	Right	cgtgacctctgcgttctgt	100
**ATF3-Raw-intron**	Left	tgcaatacatgagcgtagca	137
**ATF3-Raw-intron**	Right	gtagccgaggtaaggggaaa	137

**ATF3-Raw-control**	Left	gatcgcagctccacagatg	131
**ATF3-Raw-control**	Right	ttgaaggacgaggatatcgag	131
**ATF3-Raw-peak**	Left	ggcattacgtcttgcacaaa	149
**ATF3-Raw-peak**	Right	tgtgcgcgccatataaaat	149
**ATF3-A3-3-control**	Left	gcgacgcaaaaggcgaatta	103
**ATF3-A3-3-control**	Right	tttccgcttctggacatccc	103
**ATF3-A3-3-peak**	Left	cacagtgcgattctaacggc	5
**ATF3-A3-3-peak**	Right	caagcacaagcacagcacat	5

Supplementary Table 3: Antibodies used in the study

**Antibody**	**Source**	**Catalog No.**	**Host species**	**Concentration**
**Armadillo**	DSHB	N2 7A1	mouse	1:200
**Beta-galactosidase**	DSHB	40-1a	mouse	1:800
**Delta**	DSHB	C594.9B	mouse	1:100
**Prospero**	DSHB	MR1A	mouse	1:1000
**Phospho histone 3**	Cell signaling	9701s	rabbit	1:500
**Cleaved Drosophila Dcp-1**	Cell signaling	9578S	rabbit	1:600
**GFP**	Cell signaling	2555S	rabbit	1:500

Supplementary References:

1. Sekyrova, P., Bohmann, D., Jindra, M., Uhlirova, M. Interaction between *Drosophila* bZIP proteins Atf3 and Jun prevents replacement of epithelial cells during metamorphosis. Development. 137: 141-50 (2010).

2. Zeitlinger, J. and Bohmann, D. Thorax closure in *Drosophila*: involvement of Fos and the JNK pathway. Development. 126: 3947-56 (1999).
